# Protein O-fucosylation in *Plasmodium falciparum* ensures efficient infection of mosquito and vertebrate hosts

**DOI:** 10.1038/s41467-017-00571-y

**Published:** 2017-09-15

**Authors:** Sash Lopaticki, Annie S. P. Yang, Alan John, Nichollas E. Scott, James P. Lingford, Matthew T. O’Neill, Sara M. Erickson, Nicole C. McKenzie, Charlie Jennison, Lachlan W. Whitehead, Donna N. Douglas, Norman M. Kneteman, Ethan D. Goddard-Borger, Justin A. Boddey

**Affiliations:** 1 The Walter and Eliza Hall Institute of Medical Research, 1G Royal Parade, Parkville, VIC 3052 Australia; 2 Department of Medical Biology, University of Melbourne, Parkville, VIC 3010 Australia; 3 Department of Microbiology and Immunology, University of Melbourne at the Peter Doherty Institute for Infection and Immunity, Parkville, VIC 3010 Australia; 4 Department of Surgery, University of Alberta, Edmonton, AB Canada T6G 2E1; 5 Radboud University Medical Center, Department of Medical Microbiology, HB 6500 Nijmegen, The Netherlands

## Abstract

O-glycosylation of the *Plasmodium* sporozoite surface proteins CSP and TRAP was recently identified, but the role of this modification in the parasite life cycle and its relevance to vaccine design remain unclear. Here, we identify the *Plasmodium* protein O-fucosyltransferase (POFUT2) responsible for O-glycosylating CSP and TRAP. Genetic disruption of *POFUT2* in *Plasmodium falciparum* results in ookinetes that are attenuated for colonizing the mosquito midgut, an essential step in malaria transmission. Some POFUT2-deficient parasites mature into salivary gland sporozoites although they are impaired for gliding motility, cell traversal, hepatocyte invasion, and production of exoerythrocytic forms in humanized chimeric liver mice. These defects can be attributed to destabilization and incorrect trafficking of proteins bearing thrombospondin repeats (TSRs). Therefore, POFUT2 plays a similar role in malaria parasites to that in metazoans: it ensures the trafficking of *Plasmodium* TSR proteins as part of a non-canonical glycosylation-dependent endoplasmic reticulum protein quality control mechanism.

## Abstract (web summary)

The role of O-glycosylation in the malaria life cycle is largely unknown. Here, the authors identify a *Plasmodium* protein O-fucosyltransferase and show that it is important for normal trafficking of a subset of surface proteins, particularly CSP and TRAP, and efficient infection of mosquito and vertebrate hosts.

## Introduction

P*lasmodium* spp. lack many genes necessary for conventional N- and O-glycosylation^1, 2^ and the N-glycan-dependent protein folding quality control pathways found in most eukaryotes^3, 4^. This has fuelled debate about whether these protozoan parasites glycosylate their proteins^1, 2, 5^. Recent advances have begun to resolve this issue^6, 7^. Blood stage *Plasmodium falciparum* parasites, which cause the most severe form of human malaria, N-glycosylate proteins with Asn-linked N-acetylglucosamine or chitobiose^6^. While this minimalistic N-glycan likely plays a thermodynamic role in protein folding^8^, it remains unclear which parasite proteins are N-glycosylated and whether it occurs in other stages of the *Plasmodium* lifecycle. In contrast, O-glycosylation has only been detected outside of the blood stages: the *P. falciparum* sporozoite antigens circumsporozoite protein (CSP) and thrombospondin-related anonymous protein (TRAP) both bear an O-linked hexosyl-deoxyhexose disaccharide on their thrombospondin repeat (TSR) domains^7^.

CSP^9^ and TRAP^10, 11^ are essential for infection of the human host and abundant on the sporozoite surface, making them prime vaccine candidates. Indeed, the only malaria vaccine approved to date RTS,S/A01^12^, is based on the CSP TSR domain. Simple O-glycans can enhance antigenicity and comprise part of T-cell epitopes^13^, making this parasite glycan of great relevance to the design of next-generation malaria vaccines. However, before pursuing this idea the precise chemical nature of the O-glycan must be determined, as should its function in the malaria parasite.

The chemical identity of the *P. falciparum* O-glycan is most likely the same as the O-linked β-D-glucosyl-1,3-*α*-L-fucose disaccharide found on metazoan TSR domains^14–16^. In these systems, O-glycosylation of the cysteine-rich TSR domain occurs in the endoplasmic reticulum (ER) on correctly folded proteins at the CXX(**S/T**)C sequon in a stepwise manner: protein O-fucosyltransferase 2 (POFUT2)^17^ O-fucosylates Ser/Thr of the protein using GDP-fucose, then β-1,3-glucosyltransferase (B3GLCT)^16^ utilizes UDP-glucose to glucosylate the 3-OH of the fucose residue (Supplementary Fig. 1)^18^. This process comprises part of a non-canonical protein folding quality control mechanism^18^. Ablation of POFUT2 or B3GLCT in mammals affects folding and trafficking of proteins with TSR domains, though the extent to which this occurs varies from protein to protein^18–23^. *POFUT2* disruption in mice has an embryonic lethal phenotype^22^ while mutations in human *B3GLCT* cause Peters-Plus syndrome^18^. The identification of glycosylated TSR proteins in *P. falciparum* suggests a similar protein quality control mechanism is present in the malaria parasite, an idea supported by the observation that heterologous expression of CSP^24^ and TRAP^25^ TSR domains in mammalian cell lines yield proteins modified with the same β-D-glucosyl-1,3-α-L-fucose disaccharide.

Here, we identify and characterize the protein O-fucosyltransferase 2 (POFUT2) conserved in all *Plasmodium* spp. Genetic disruption of *POFUT2* in *P. falciparum* results in attenuation of both ookinete and sporozoite infection of their respective mosquito and vertebrate hosts. The defects are attributable to destabilization and incorrect trafficking of proteins with TSRs. This suggests that POFUT2 plays an important role in parasite transmission to mosquitoes and infection of the human host by ensuring trafficking of TSR proteins following glycosylation in the parasite ER.

## Results

### In vitro characterization of *Plasmodium* POFUT2

To investigate whether malaria parasites encode a POFUT2, a BLAST search of the *P. falciparum* genome using *Homo sapiens* POFUT2 (CAC24557.1)^26^ as a search term led to the identification of PF3D7_0909200 as the putative malarial POFUT2 enzyme. Highly homologous syntenic orthologs were present across the *Plasmodium* genus (Supplementary Fig. 2). The putative *P. falciparum* and *P. vivax* (PVX_098900.1) POFUT2 share considerable sequence similarity with *H. sapiens* and *Caenorhabditis elegans* (NP_001255070.1) POFUT2 and retain the catalytic residues of these enzymes (Supplementary Fig. 3). A homology model constructed from the *P. falciparum* POFUT2 sequence has a very similar predicted structure to *H. sapiens* and *C. elegans* POFUT2 (Supplementary Fig. 4A), sufficient to allow the GDP-fucose and TSR domain substrates to be docked into the model to reveal an alignment of catalytic residues and substrates that is reminiscent of a Michaelis complex (Supplementary Fig. 4B, C)^26, 27^.

**Fig. 1:**
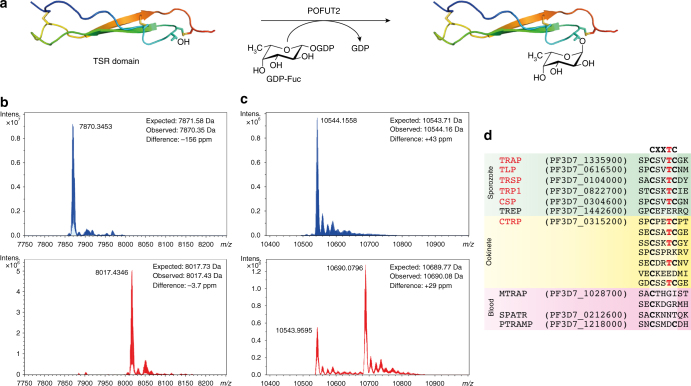
O-Fucosylation of TSR domains by POFUT2 in *P. falciparum*. **a** O-Fucosylation of TSR domains by GDP-fucose, as catalyzed by POFUT2, (illustration generated using 4HQO)^25^. **b** Deconvoluted intact ESI mass spectrum of recombinant *P. falciparum* TRAP TSR domain treated with GDP-fucose in the absence (*blue*) and presence (*red*) of *P. vivax* POFUT2. **c** Deconvoluted intact ESI mass spectrum of recombinant *P. falciparum* CSP TSR domain treated with GDP-fucose in the absence (*blue*) and presence (*red*) of *P. vivax* POFUT2. **d** Multiple sequence alignment of all TSR domain sequences from *P. falciparum* revealing the proteins that are likely to be O-fucosylated (*red*) and in what parasite stage they are expressed

We sought to recombinantly express putative POFUT2 and TSR domains from the *Plasmodium* genus to demonstrate enzymatic activity in vitro and support the notion that the deoxyhexose observed previously by MS on *P. falciparum* CSP and TRAP^7^ is in fact L-fucose and is localized on the threonine of the CXX**T**C sequon. All attempts to express *P. falciparum* and *P. vivax* POFUT2 in *Escherichia coli* and *Pichia pastoris* were unsuccessful. Recombinant *P. vivax* POFUT2 was obtained by secretion as a SUMO fusion protein from Sf21 insect cells using a baculovirus expression system (Supplementary Fig. 5), though this strategy failed for *P. falciparum* POFUT2. The TSR domains of *P. falciparum* TRAP and CSP were expressed in *E. coli* as a GST fusion protein and the GST tags removed using HRV C3 protease (Supplementary Fig. 5). Recombinant *P. vivax* POFUT2 and GDP-fucose were incubated with each TSR domain (Fig. 1a) and analyzed by intact electrospray ionisation mass spectrometry (ESI-MS) to reveal a mass shift of + 146 for both TRAP (Fig. 1b) and CSP (Fig. 1c), indicating the addition of a single L-fucose to the proteins. This mass shift was not observed in the absence of *P. vivax* POFUT2. LC-MS/MS analysis of GluC-digested samples of the O-fucosylated TRAP enabled the localization of this glycosylation to the threonine residue of the CXX**T**C motif (Supplementary Fig. 6). This confirmed that *Plasmodium* parasites possess a conserved syntenic POFUT2 capable of O-fucosylating TSR domains on the canonical serine/threonine residue of the CXX(**S/T**)C motif.

We proceeded to inspect the sequence of every *P. falciparum* protein with a TSR domain for this O-fucosylation sequon (Fig. 1d) to and found that, in addition to CSP and TRAP, potential POFUT2 substrates include: circumsporozoite- and TRAP-related protein (CTRP), expressed in ookinetes^28, 29^, as well as TRAP-like protein (TLP)^30^, thrombospondin-related sporozoite protein (TRSP)^31^ and thrombospondin-related protein 1 (TRP1)^32^ from the sporozoite stages. No blood stage proteins with a TSR domain possessed the consensus site, suggesting that O-fucosylation was not important to this stage of the parasite’s life cycle.

### *Plasmodium* POFUT2 localizes to the ER

To identify the subcellular location of POFUT2 in *P. falciparum*, transgenic NF54 parasites were produced in which the *POFUT2* gene encoded triple hemagglutinin (HA) epitopes in-frame at the C-terminus such that expression was still driven by the endogenous promoter (Supplementary Fig. 7A). Integration of the HA epitope cassette was validated by Southern blot (Supplementary Fig. 7B) and POFUT2-HA expression confirmed by immunoblot using anti-HA antibodies. POFUT2-HA migrated as a single species of circa 60 kDa in asexual parasites, consistent with a predicted mass of 59 kDa (Supplementary Fig. 7C). Immunofluorescence microscopy revealed puncta of POFUT2-HA expression that co-localized with plasmepsin V^33^, consistent with an ER localization in *P. falciparum* (Supplementary Fig. 7D). The punctate distribution pattern within the ER suggests that POFUT2-HA localizes within sub-domains of the ER, the presence of which has been described previously^34^. Detection of POFUT2 expression in asexual parasites is consistent with reports that GDP-fucose is biosynthesized in blood stage *P. falciparum* parasites^35^, though it does not appear to be essential^36^. It is unclear what, if any, protein(s) might be O-fucosylated by POFUT2 in the blood stage (Fig. 1d).

### Generation of POFUT2-deficient *P. falciparum*

To study the function of POFUT2 in *P*. *falciparum*, isogenic NF54 parasites were generated in which the *POFUT2* locus was excised by double cross over homologous recombination (Supplementary Fig. 8A). Two independent clones of Δ*POFUT2* parasites (D3 and G8) were generated by limiting dilution and validated by Southern blot analysis (Supplementary Fig. 8B). Both mutant clones developed within erythrocytes at the same rate as NF54 parasites, indicating that *POFUT2* is not essential for asexual blood stage growth (Supplementary Fig. 8C), in agreement with GDP-fucose being dispensable^36^ and the absence of predicted substrates in this stage (Fig. 1d). The Δ*POFUT2* parasites were differentiated into gametocytes and no significant difference in stage V gametocytemias were observed compared to NF54, demonstrating that *POFUT2* is not essential for gametocytogenesis (Supplementary Fig. 8D).

### POFUT2 facilitates *P. falciparum* infection of the mosquito

**Fig. 2:**
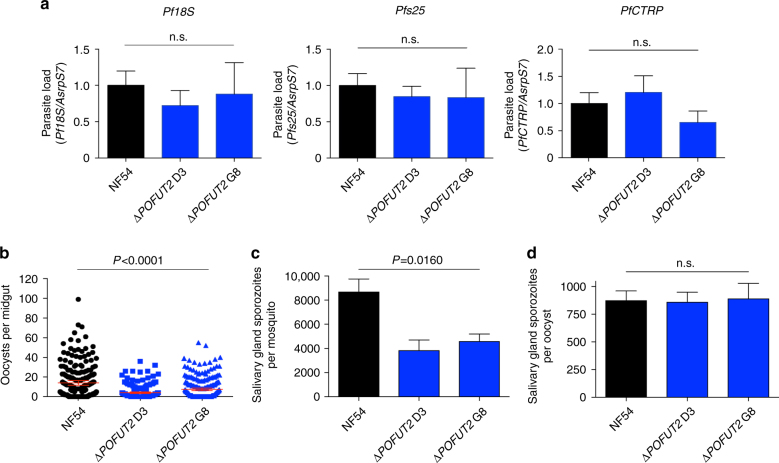
POFUT2 is important for *P. falciparum* transmission to *Anopheles stephensi* mosquitoes. **a** Parasite load in mosquito midguts 27 h post-bloodmeal, measured by qRT-PCR of transcripts for *Pf18S* (total parasites)*, Pfs25* (gametes, zygotes, ookinetes), and *PfCTRP* (ookinetes) relative to *Anopheles stephensi* ribosomal protein, rps7 (AsrpS7). No significant differences were observed relative to NF54 for *Pf18S* (*P* = 0.4973), *Pfs25* (*P* = 0.3513), and *PfCTRP* (*P* = 0.2547). **b** Oocyst counts per mosquito midgut 7 days post-bloodmeal. Data are mean ± 95% confidence interval from three independent experiments. **c** Salivary gland sporozoite count per mosquito 17 days post-bloodmeal. **d** Salivary gland sporozoite count divided by oocyst count (*P* = 0.9945). Data is the mean ± S.E.M. from three independent experiments. *P*-values are for both mutant clones compared to NF54, calculated using the Kruskal–Wallis one-way ANOVA

To examine the function of POFUT2 in other *P. falciparum* lifecycle stages, mature gametocytes were fed to female *Anopheles stephensi* mosquitoes by membrane feeding. Parasite load and differentiation within the mosquito was determined by real-time quantitative reverse-transcription PCR (qRT-PCR) of infected midguts 27 h post-bloodmeal. Quantification of *Pf18S* transcripts revealed that total parasite load in the mosquitoes did not differ between NF54 and Δ*POFUT2* parasites (Fig. 2a). *Pfs25* transcripts, which are produced by gametes, zygotes and ookinetes^37^ and *CTRP* transcripts, which are expressed in ookinetes^28^, were also statistically equal between parasite strains. This implies that POFUT2 is not essential for formation of ookinetes within the mosquito. However, the number of oocysts developing at the basal lamina of mosquito midguts was reduced for both Δ*POFUT2* clones relative to NF54 (range 63–87% reduction; *P* < 0.0001 using the Kruskal–Wallis one-way analysis of variance (ANOVA)) (Fig. 2b). This indicates that POFUT2 is required for normal infection of the mosquito vector by *P. falciparum* ookinetes. The sole predicted TSR protein expressed in ookinetes is CTRP (Fig. 1d), which is essential for ookinete motility and invasion of the midgut^28, 29^. Our results are, therefore, consistent with perturbed glycosylation and function of CTRP, resulting in defective midgut invasion. *P. falciparum* ookinetes could not be successfully cultured in vitro to confirm this by proteomic analysis.

### POFUT2 supports *P. falciparum* sporozoite infectivity and fitness

**Fig. 3:**
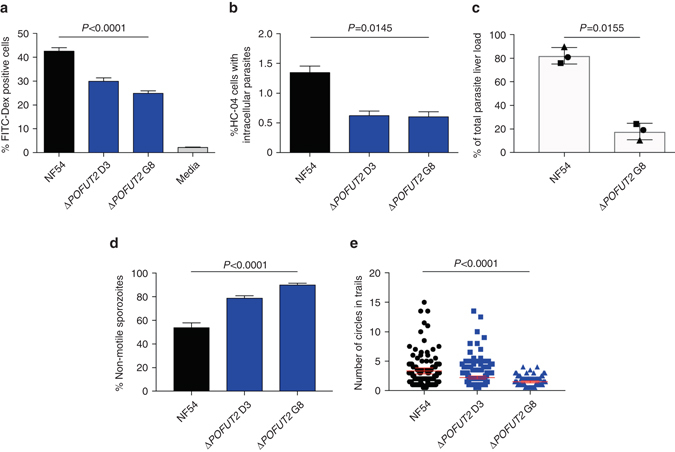
POFUT2 facilitates *P. falciparum* liver infection. **a** Percentage of traversed (FITC-dextran positive) human HC-04 hepatocytes by salivary gland sporozoites. **b** Percentage of HC-04 cells with intracellular parasites 24 h after addition of sporozoites to cells. Data is the mean ± S.E.M. from three (**a**) and two (**b**) independent experiments. **c** Parasite liver load measured by qPCR showing the fitness of Δ*POFUT2* versus parental NF54 sporozoites following coinfection of three humanized chimeric liver mice. Each *symbol* corresponds to the same coinfected mouse. Data are mean ± S.E.M. **d** Percent of sporozoites that are non-motile in a two-dimensional gliding motility assay. **e** Number of circles per trail produced by gliding sporozoites (non-motile parasites removed). Data in **d**, **e** is mean ± S.E.M. or 95% confidence interval, respectively, from two independent experiments. *P*-values are for both mutant clones compared to NF54, calculated using the Kruskal–Wallis one-way ANOVA, except panel **c**, which compared one mutant clone to NF54 in each of three mice using the paired *t*-test

To investigate the function of POFUT2 in sporozoites, parasites were propagated through mosquitoes and dissected from salivary glands. Mosquitoes infected with Δ*POFUT2* parasites harboured 45–55% fewer sporozoites in their salivary glands, depending on the clone, than mosquitoes infected with the NF54 parental line (*P* = 0.0160 using the Kruskal–Wallis one-way ANOVA; Fig. 2c). Since Δ*POFUT2* parasites produce fewer oocysts, this result was expected. When standardizing for oocysts, the number of salivary gland sporozoites was not different (Fig. 2d), suggesting that POFUT2 function may not be critical for *P. falciparum* maturation in oocysts or salivary gland invasion. Next, we assessed whether sporozoites in the salivary glands were infectious. Cell traversal activity is required for liver infection and was measured by incubation of sporozoites with human HC-04 hepatocytes in the presence of FITC-dextran and quantifying dextran-positive cells^38, 39^. Δ*POFUT2* parasites were reduced for cell traversal by 30–42%, depending on the mutant clone (*P* < 0.0001 using the Kruskal–Wallis one-way ANOVA) (Fig. 3a). The ability for sporozoites to invade hepatocytes, which is critical for liver infection, was investigated by quantifying the number of parasites inside HC-04 hepatocytes 24 h post-addition of sporozoites to cells. This revealed a strong defect in the number of intracellular Δ*POFUT2* parasites (*P* = 0.0145 using the Kruskal–Wallis one-way ANOVA) (Fig. 3b), consistent with a defect in invasion into the cells. To examine the effect of POFUT2 activity on parasite fitness in vivo, coinfection experiments were performed in which an equal inoculum of NF54 and mutant sporozoites was mixed and injected intravenously into humanized chimeric liver mice^40^. Loss of POFUT2 function resulted in a severe fitness cost, as demonstrated by an approximate 80% reduction in parasite liver load compared to NF54 parents (*P* = 0.0155 using the paired *t*-test; Fig. 3c). Therefore, POFUT2 activity is important for liver infection by *P. falciparum* sporozoites.

Gliding locomotion is obligatory for infectivity of sporozoites^10, 41^. We, therefore, assessed whether POFUT2 plays a role in gliding motility by measuring sporozoite trails on a solid substrate^42^. Loss of POFUT2 function caused a reduction in gliding motility, reflected by an increase in non-motile sporozoites (Fig. 3d) and concomitant decrease in the number of trail circles produced by Δ*POFUT2* sporozoites that could glide (Fig. 3e) (*P* < 0.0001 using the Kruskal–Wallis one-way ANOVA). Therefore, POFUT2 is required for normal gliding motility in *P. falciparum*, which provides a mechanistic explanation for why mutant sporozoites were less infective in vitro and less fit in vivo.

### POFUT2 assists stabilization and trafficking of TSR proteins

**Fig. 4:**
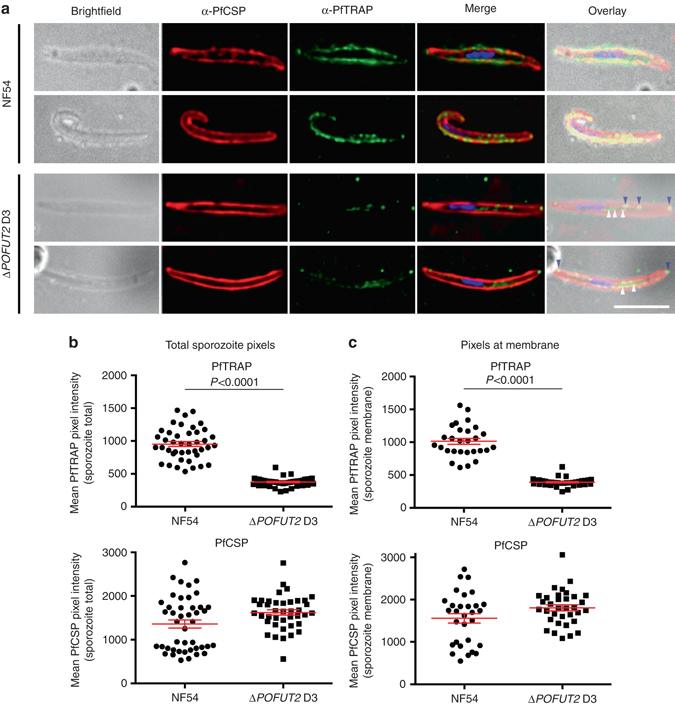
POFUT2 plays a role in TSR protein trafficking in *P. falciparum*. **a** Immunofluorescence microscopy of NF54 and Δ*POFUT2* salivary gland sporozoites showing the localization of CSP (*red*), TRAP (*green*), and dsDNA (*blue*). *Purple arrow*, TRAP at the sporozoite membrane; *white arrow*, TRAP internal to parasite. *Scale* 5 μm. **b** Total sporozoite pixel intensity for PfTRAP and PfCSP. **c** Pixel intensity for PfTRAP and PfCSP at the sporozoite membrane only. Data in **b**, **c** is the mean ± S.E.M. from two independent experiments. In panel **b**, a subtle increase in total PfCSP pixels was observed in Δ*POFUT2* D3 relative to NF54 (*P* = 0.0360) but no difference was observed at the sporozoite membrane (*P* = 0.1083) in panel **c**. *P*-values are for one mutant clone compared to NF54, calculated using the Mann–Whitney test

**Fig. 5:**
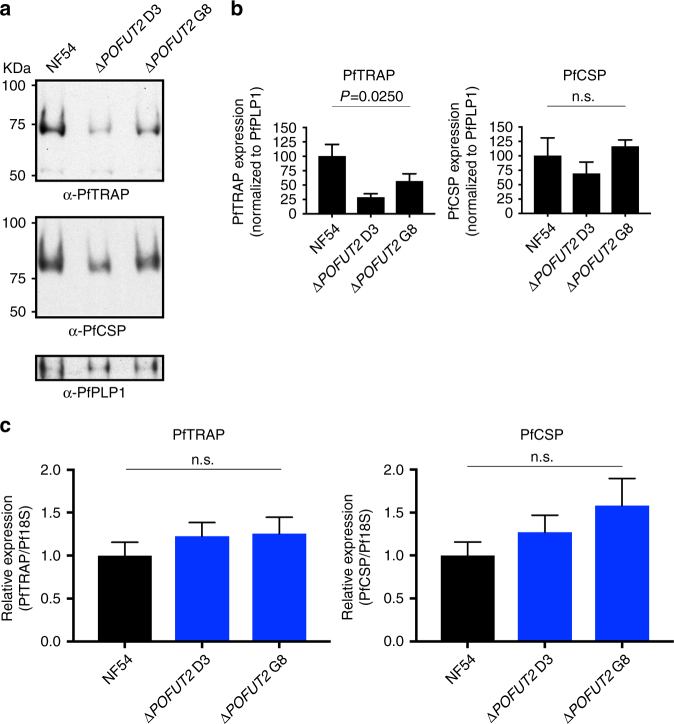
POFUT2 stabilizes TSR proteins in *P. falciparum*. **a** Western blot analysis of 30,000 salivary gland sporozoites per lane using antibodies to PfTRAP and PfCSP. Anti-PfPLP1 was used as a loading control. The same blot was probed consecutively with each antibody. **b** Densitometry of PfTRAP and PfCSP levels in sporozoites measured by immunoblotting and standardized to the PfPLP1 loading control. Data are mean ± S.E.M. and pooled from three independent immunoblots. **c** Abundance of *PfTRAP* and *PfCSP* mRNA transcripts in salivary gland sporozoites relative to *Pf18S*, measured by qRT-PCR. No differences were observed for either Δ*POFUT2* clone compared to NF54 for *PfTRAP* (*P* = 0.5003) and *PfCSP* (*P* = 0.3104) mRNA. Data is the mean ± S.E.M. of four independent experiments. *P*-values are for both mutant clones compared to NF54, calculated using the Kruskal–Wallis one-way ANOVA

Given the important function of POFUT2 in protein quality control in metazoans^18^, we examined whether proteins were destabilized or trafficked differently following loss of POFUT2 activity. Since the two TSR proteins reported to be O-fucosylated in sporozoites are CSP and TRAP^7^, we investigated these two proteins by immunofluorescence microscopy using antibodies directed to these proteins (Fig. 4a). While we observed no decrease in CSP pixel intensity between NF54 and Δ*POFUT2* sporozoites, the total TRAP pixel intensity was dramatically reduced in POFUT2-deficient sporozoites (*P* < 0.0001 using the Mann–Whitney test) (Fig. 4b). Furthermore, the intensity of TRAP pixels at the sporozoite membrane was also significantly reduced in Δ*POFUT2* parasites (*P* < 0.0001 using the Mann–Whitney test) and TRAP was commonly observed inside parasites, indicating that trafficking to the sporozoite membrane was impaired by loss of POFUT2 function (*P* < 0.0001 using the Mann–Whitney test) (Fig. 4c). Analysis of protein expression levels by immunoblotting indicated that levels of TRAP (*P* = 0.0250) but not CSP (*P* = 0.3571) (*P*-values were determined using the Kruskal–Wallis one-way ANOVA) were reduced in Δ*POFUT2* sporozoites (Fig. 5a, b). To confirm that the difference was due to protein destabilization rather than decreased gene expression, qRT-PCR was performed on sporozoites. This demonstrated no significant difference in the relative abundance of Pf*TRAP* or Pf*CSP* messenger RNA (mRNA) transcripts between NF54 and Δ*POFUT2* sporozoites (Fig. 5c). Therefore, POFUT2 activity is required for the stabilization and trafficking of some TSR proteins in *P. falciparum*, although TRAP is more dependent than CSP on O-fucosylation for stabilization and trafficking (Fig. 5). This may be because the TSR domain of CSP possesses just two disulfides, while that of the TRAP domain contains three and is likely more susceptible to misfolding and subsequent degradation due to erroneous disulfide bond formation.

## Discussion

The recent discovery of O-glycosylation on CSP and TRAP in *P. falciparum* sporozoites represents a very important advance in our understanding of *Plasmodium* glycobiology^7^ and complements recent work on the biosynthesis of sugar nucleotides in *P. falciparum* ^35, 36^. We have significantly built on these findings by identifying the ER-resident enzyme responsible for O-glycosylation in *P. falciparum* and confirming that POFUT2 is a fucosyltransferase that modifies parasite TSR domains specifically with L-fucose at the conserved serine/threonine residue within the CXX(**S/T**)C sequon. Our study also demonstrates that this enzyme plays an important role in stabilizing TRAP in sporozoites and possibly other TSR proteins, such as CTRP in ookinetes, to ensure successful infection of the mosquito vector and human liver cells.

It is not clear why POFUT2 and GDP-fucose are produced in the blood stages of *P. falciparum*. While MTRAP, SPATR, and PTRAMP are expressed in asexual stages and contain TSRs, they lack the critical CXX**S/T**C O-fucosylation sequon suggesting they are not fucosylated. Nonetheless, our study indicates that POFUT2 is not essential for parasite growth in the asexual or sexual blood stages. This is supported by recent studies showing that disruption of genes involved in GDP-fucose biosynthesis, GDP-mannose 4,6-dehydratase and GDP-L-fucose synthase, had no impact on asexual or sexual development^36^ and are also expressed in mosquito stages^43, 44^.

Conversely, O-fucosylation is important in parasite stages that develop within the mosquito. Our analyses of infected mosquitoes suggest that O-fucosylation is not essential for *P. falciparum* differentiation into ookinetes but the formation of fewer oocysts implies it is important for ookinete infection of mosquito midgut epithelial cells. An obvious role for POFUT2 in ookinetes is the O-fucosylation of five of the seven TSR domains of the essential motility-associated adhesin CTRP (Fig. 1d)^28, 29^. Given that POFUT2 proteins play a key role in protein quality control and trafficking in metazoans^18^, it is tempting to speculate that CTRP is reliant on POFUT2 for stabilization and trafficking. A dearth of appropriate antibodies and methods for the production of large quantities of *P. falciparum* ookinetes has prevented us from experimentally confirming this hypothesis, which might be better examined in a *P. berghei* system using in vitro ookinete culture^45^.

POFUT2-deficient parasites produced oocysts, albeit less than wild type, and this provided the opportunity to study sporozoites. POFUT2 mutants produced fewer salivary gland sporozoites within the mosquito than their NF54 parents, as expected based on the formation of fewer oocysts. When the number of salivary gland sporozoites was standardized for oocysts, no defect was observed suggesting the levels of CSP, TRP1, and TRAP remaining in Δ*POFUT2* sporozoites was sufficient to allow their important functions in sporulation, oocyst egress and salivary gland invasion^9, 10, 32, 46^. Indeed, our analysis of protein levels in sporozoites showed that TSR proteins are impacted differently by the loss of POFUT2 function, which is reminiscent of observations made in metazoans, where some proteins are impacted far more than others^18^. We could not demonstrate statistically significantly destabilization of CSP but TRAP was substantially destabilized by loss of POFUT2 activity, though not fully degraded. Given the essential roles of CSP in so many aspects of sporozoite biology, it is plausible that its TSR domain (with only two disulphide bonds) has evolved to be more stable than the TRAP TSR domain (with its three disulphide bonds) and classical TSR topology^25^.

Our study has also shown that POFUT2 function extends to sporozoite interactions with human hepatocytes both in vitro and in humanized mice with chimeric livers and is therefore implicated in *P. falciparum* transmission to humans. Δ*POFUT2* sporozoites were defective for cell traversal activity, invasion of human hepatocytes and for fitness of sporozoites in vivo. These phenotypes can be attributed to functional destabilization of TRAP and CSP but may also include other TSR proteins that mediate sporozoite motility and invasion of hepatocytes, such as TLP and TRSP, respectively^10, 30, 31, 47^. Our observations of reduced gliding motility of POFUT2-deficient sporozoites strongly support this hypothesis. Further study is needed to verify that these other TSR proteins are O-glycosylated and reliant on POFUT2 for correct folding and trafficking.

The discovery of O-glycosylation in *Plasmodium* parasites is an important advance in our understanding of parasite biology and the significant defects we describe illustrate the relevance of this modification to propagation of the parasite through its lifecycle. Loss of POFUT2 function abrogated protein stabilization and impaired protein trafficking, impacting on both the transmission of ookinetes to mosquitoes and the infectivity of sporozoites. This demonstrates that the ER protein glycosylation process in malaria parasites confers a significant survival advantage and is important for malaria transmission. It appears unlikely that O-fucosylation is dispensable to parasites, and so incorporation of these glycans into vaccines based on CSP, TRAP, CTRP, and possibly other TSR proteins are well worth investigating. The production of such antigens should be relatively straight forward, since mammalian glycosyltransferases can recognize and modify heterologously expressed *Plasmodium* TSR domains^24, 25^.

## Methods

### POFUT2 enzyme assay

Details of recombinant protein expression are provided in the Supplementary Methods. Reactions (10 μl total volume) containing recombinant *P. falciparum* TRAP TSR (10 μM), recombinant *P. vivax* POFUT2 (10 ng), GDP-Fuc (50 μM), MgCl_2_ (5 mM) in buffer (20 mM Tris, 150 mM NaCl, pH 7.4) were incubated for 16 h at 25 °C. Negative controls included reactions without *P. vivax* POFUT2. Samples were snap-frozen and stored at –80 °C until analysis by mass spectrometry, as detailed in the Supplementary Methods.

### Parasite maintenance

*P. falciparum* NF54 asexual stages were maintained in human type O-positive erythrocytes (Melbourne Red Cross) in RPMI-HEPES supplemented with 10% heat-inactivated human serum (Melbourne Red Cross), at 37 °C. Gametocytes for transmission to mosquitoes were generated using the “crash” method^48^ using daily media changes.

### Transgenic parasites

*P. falciparum* NF54 (kindly provided by the Walter Reid Army Institute of Research) was used to generate all transgenic parasites. Details for cloning, transfection, selection, and validation of transgenic lines are provided in the Supplementary Methods.

### Blood stage growth assay

Synchronized trophozoite stage parasites were added to erythrocytes to 0.2% parasitemia, 1% hematocrit. Starting parasitemia was confirmed by flow cytometry (FACSCalibur; BD) using ethidium bromide staining (1:1000 dilution in phosphate buffered saline (PBS))^33^ and final parasitemia was determined 96 h later. For each line, triplicate samples of 50,000 cells were counted in each of three independent experiments. Growth was expressed as a percentage of NF54.

### Mosquito infection and analysis of parasite development

Five- to seven-day old female *Anopheles stephensi* mosquitoes (this strain originally provided by M. Jacobs-Lorena, Johns Hopkins University) were fed on asynchronous gametocytes, diluted to 0.3–0.6 % stage V gametocytemia, via water-jacketed glass membrane feeders. Mosquitoes were sugar starved for 2 days post-bloodmeal to enhance the population for blood-fed mosquitoes. Surviving mosquitoes were provided 5% glucose ad libitum via paper/cotton wicks or water wicks and sugar cubes. Oocyst numbers were obtained from midguts dissected from cold-anesthetized and ethanol-killed mosquitoes 7 days post-bloodmeal and stained with 0.1% mercurochrome. Salivary glands were dissected from mosquitoes (day 16–20 post-bloodmeal), crushed using pestle and then glass wool filtered to obtain sporozoites used in subsequent assays. Mosquito bloodmeal bolus were isolated 27 h post-feed from mosquitoes to check for the presence of ookinetes via qRT-PCR (see Supplementary Table 1). Briefly, mosquitoes were cold anesthetized and ethanol killed. Midguts were carefully dissected and frozen immediately on dry ice. RNA was purified using TRI Reagent (Sigma) and complementary DNA (cDNA) prepared using a SensiFast cDNA synthesis kit (Bioline) according to the manufacturers’ instructions and qRT-PCR performed using a LightCycler 480 (Roche). All oligonucleotides used in this study are listed in Supplementary Table 1.

### Immunofluorescence microscopy

Asexual stages were fixed in ice-cold methanol and probed with rat anti-HA (1:500; Roche 3F10) and rabbit anti-plasmepsin V (PMV) (1:1000) antibodies^33^ in 3% bovine serum albumin (BSA)/PBS. Salivary gland sporozoites were air-dried on slides, fixed in 4% paraformaldehyde and permeabilized in 0.1% triton X-100. Primary antibodies (mouse monoclonal anti-CSP (2A10); 1:2000^49^) (rabbit anti-TRAP 1:500^50^) were diluted in 3% BSA/PBS. Secondary antibodies were goat anti-rabbit 594 and anti-mouse or -rat Alexa 488 (1:1000; Invitrogen).Micrographs were acquired on a Deltavision Elite microscope (Applied Precision) using an Olympus 100Å~/1.42 PlanApoN objective equipped with a Coolsnap HQ2 CCD camera as Z-stacks. Images were deconvolved and presented as maximum intensity projections. For quantification of mean pixel intensity, Z-stacks of *n* ≥ 30 sporozoites per condition per experiment were captured using the same exposure settings on the Deltavision system to allow quantitative analysis between different samples. The analysis was performed with a custom FIJI macro. The sporozoite was segmented by filtering and thresholding the sum of all three fluorescence channels, and the membrane area determined by stepping in from the edge of the filtered segmented parasite by three pixels. Mean pixel intensity measurements for TRAP and CSP were performed on each of these regions in the 488 and 594 channels, respectively, and statistically compared in duplicate independent experiments.

### Sporozoite gene expression analysis

Sporozoites were dissected from salivary glands on day 17 or 18 post-blood meal. RNA was purified using TRI Reagent (Sigma) and cDNA prepared using a SensiFast cDNA synthesis kit (Bioline) according to the manufacturers’ instructions and qRT-PCR performed using a LightCycler 480 (Roche) with oligonucleotides in Supplemetary Table 1.

### Immunoblotting

Proteins were separated through 4–12% Bis-Tris polyacrylamide gels (Invitrogen), transferred to nitrocellulose membrane and probed with primary antibodies: rat anti-HA 1:500 (Roche 3F10), mouse monoclonal anti-CSP (2A10) 1:9000^49^, rabbit anti-TRAP 1:2000;^50^ rabbit anti-PLP1 (1:200)^39^, rabbit anti-Aldolase (1:4000)^51^ followed by horse radish peroxidase-conjugated secondary antibodies (1:1000 (mouse) and 1:4000 (rabbit); Cell Signaling Technology) and viewed by enhanced chemiluminescence (Amersham).

### Hepatocyte culturing

HC-04 hepatocytes^52^ were maintained on Iscove’s Modified Dulbecco’s Medium (IMDM), supplemented with 5% heat-inactivated fetal bovine serum (FBS) at 37 °C in 5% CO_2_. Cells were split 1:6 every 2–3 days once they reached ~90% confluency.

### Cell traversal assay

Cell traversal was measured using a cell-wounding assay^39, 53^. HC-04 hepatocytes (5 × 10^4^) were seeded into each well of a 48-well plate (Corning, Sigma Aldrich) coated with rat tail collagen. After 24 h, wells were seeded with 5 × 10^4^ sporozoites for 2.5 h in the presence of 1 mg ml^−1^ FITC-labeled dextran (10,000 MW, Sigma Aldrich). Cells were trypsinized to obtain a single cell suspension for FACS analyses. For each condition, triplicate samples of 10,000 cells were counted by FACS in each of the three independent experiments.

### Hepatocyte invasion assay

HC-04 cells (5 × 10^4^) were seeded onto rat tail collagen-coated coverslips in 24-well plates using Dulbecco's modified Eagle medium without glucose (Life Technologies, 11966-025), supplemented with 1 mM sodium pyruvate (Life Technologies, 11360-070); 1% FBS (Cellgro,35-010-CV); 1 × Pen/Strep (Corning, 30-001-Cl); 1 × MEM non-essential amino acids without l-glutamine (Sigma-Aldrich, M5550); and 1:500 dilution of Lipid Mixture 1, Chemically Defined (Sigma-Aldrich, L0-288)^39, 54^. Sporozoites (5 × 10^4^) were added to the cells 12 h later and incubated for 24 h. Media was replaced after 3 h and the assay continued on for a further 21 h (to give an invasion assay of 24 h). Coverslips were fixed in 4% paraformaldehyde for 20 min at RT and then processed as described^54^. Sporozoites were detected by immunofluorescence staining using mouse monoclonal antibodies against CSP (1:2000), anti-mouse Alexa-488 (1:1000), and anti-mouse Alexa 594 (1:1000). Multiple images were taken at 200 × magnification (Axio observer). A minimum of 270 fields with approximately 10,000 HC-04 cells were counted and the percentage of cells with intracellular sporozoites was calculated from this data set. For each condition, duplicate samples were manually counted in each of two independent experiments.

### Sporozoite gliding assay

Gliding assays were performed as described previously^40, 42^ with some minor exceptions. Eight-well chamber slides (Thermo Fisher Scientific 154534) were coated with CSP antibodies (1:1000 in PBS). Twenty thousand salivary gland sporozoites were seeded into each well and allowed to glide for 60 min at 37 °C in 5% CO_2_ in IMDM supplemented with 10% heat-inactivated human serum. Samples were fixed with 4% paraformaldehyde at 37 °C for 20 min. Primary anti-PfCSP was applied followed by goat anti-mouse Alexa 488 at (both antibodies were 1:1000 in 3% BSA). Sporozoites and trails were viewed on a Deltavision Elite microscope (Applied Precision) using an Olympus 163x/1.42 PlanApoN objective equipped with a Coolsnap HQ2 CCD camera. A total range of 220 (NF54) to 840 (Δ*POFUT2*) sporozoites were counted for each condition across two independent experiments.

### Humanized mice production, infection, and processing

uPA ^+ / +^ -SCID mice (University of Alberta) were housed in a virus- and antigen-free facility supported by the Health Sciences Laboratory Animal Services at the University of Alberta and cared for in accordance with the Canadian Council on Animal Care guidelines. All protocols involving mice were reviewed and approved by the University of Alberta Health Sciences Animal Welfare Committee and the Walter and Eliza Hall Institute of Medical Research Animal Ethics Committee. uPA ^+ / +^ -SCID mice at 5–14 days old (2 male, 1 female) received 10^6^ human hepatocytes (cryopreserved human hepatocytes were obtained from BioreclamationIVT—Baltimore MD) by intrasplenic injection and engraftment was confirmed 8 weeks post-transplantation by analysis of serum human albumin^39, 55^. An inoculum of 4.0 × 10^5^ *P. falciparum* NF54 sporozoites and 4.0 × 10^5^ Δ*POFUT2* G8 sporozoites freshly isolated from mosquito salivary glands were mixed and injected by intravenous tail injection into each of three humanized mice, as previously described^40^. Livers were obtained 6 days post-infection from CO_2_-ethanized mice and individual lobes were cut as described^56^, pooled and emulsified into a single cell suspension and flash frozen in liquid nitrogen for subsequent genomic DNA (gDNA) extraction.

### Measuring exoerythrocytic development in humanized mice

To quantify parasite load in the chimeric livers, gDNA was isolated from the single cell liver suspensions and Taqman probe-based qPCRs were performed as previously described^39, 56, 57^. To specifically differentiate NF54 from Δ*POFUT2* genomes from the same mouse samples, the following oligonucleotides were used. For NF54 genomes: POFUT2hm_F and POFUT2hm_R, which bind internal to the *POFUT2* gene in NF54 but do not amplify a product using Δ*POFUT2* parasites. For Δ*POFUT2* genomes: hDHFRhm_F and hDHFRhm_R, which bind in the hDHFR cassette in Δ*POFUT2* parasites but do not amplify a product using NF54. Human and mouse genomes were quantified using oligonucleotides specific for prostaglandin E receptor 2 (PTGER2) from each species, as described previously^57^. Sequences of primers used are provided in Supplementary Table 1. All probes were labeled 5′ with the fluorophore 6-carboxy-fluorescein (FAM) and contain a double-quencher that includes an internal ZEN™ quencher and a 3′ Iowa Black® quencher from IDT. The following probes were used:

POFUT2 5′FAM AATGTTAAT/ZEN/AGGTTCAAACAATTTTG-3IABkFQ,

hDHFR FAM/TAAACTGCA/ZEN/TCGTCGCTGTG/3IABkFQ,

hPTGER2 FAM/TGCTGCTTC/ZEN/TCATTGTCTCG/3IABkFQ,

mPTGER2 FAM/CCTGCTGCT/ZEN/TATCGTGGCTG/3IABkFQ.

Standard curves were prepared by titration from a defined number of DNA copies for *P. falciparum* NF54, Δ*POFUT2*, human and mouse controls. PCRs were performed on a Roche LC80 using LightCycler 480 Probe Master (Roche).

### Statistics

Statistical analyses were performed using the Kruskal–Wallis one-way ANOVA to compare two mutant clones to NF54 throughout this study. The Mann–Whitney test was used to compare one mutant clone to NF54 (sporozoite pixel analyses) and the paired *t*-test was employed to evaluate fitness of one mutant clone versus NF54 in each of three humanized mice. Analyses were performed using Graphpad Prism 6.

### Ethics statement

All experimental protocols involving humanized mice were conducted in strict accordance with the recommendations in the National Statement on Ethical Conduct in Animal Research of the National Health and Medical Research Council and were reviewed and approved by the Walter and Eliza Hall Institute of Medical Research Animal Ethics Committee (AEC2014.030). All experiments involving the use of human erythrocytes and the HC-04 human hepatocyte cell line were reviewed and approved by the Walter and Eliza Hall Institute of Medical Research Human Research Ethics Committee (HREC 86/17 and 15/06.

### Data availability

All data supporting the findings of this study are available within the article and its Supplementary Information files, or are available from the corresponding authors upon request.

## Electronic supplementary material

Supplementary Information
