# Supplementary material for: Protein O-fucosylation in Plasmodium falciparum ensures efficient infection of mosquito and vertebrate hosts
Source: Nat Commun. 2017 Sep 15;8:561. doi: 10.1038/s41467-017-00571-y (PMC5601480; doi:10.1038/s41467-017-00571-y)
Supplement: Supplementary file 1 — Supplementary Information [file 41467_2017_571_MOESM1_ESM.pdf]

File name: Supplementary Information
Description: Supplementary Figures, Supplementary Table, Supplementary Methods and Supplementary References

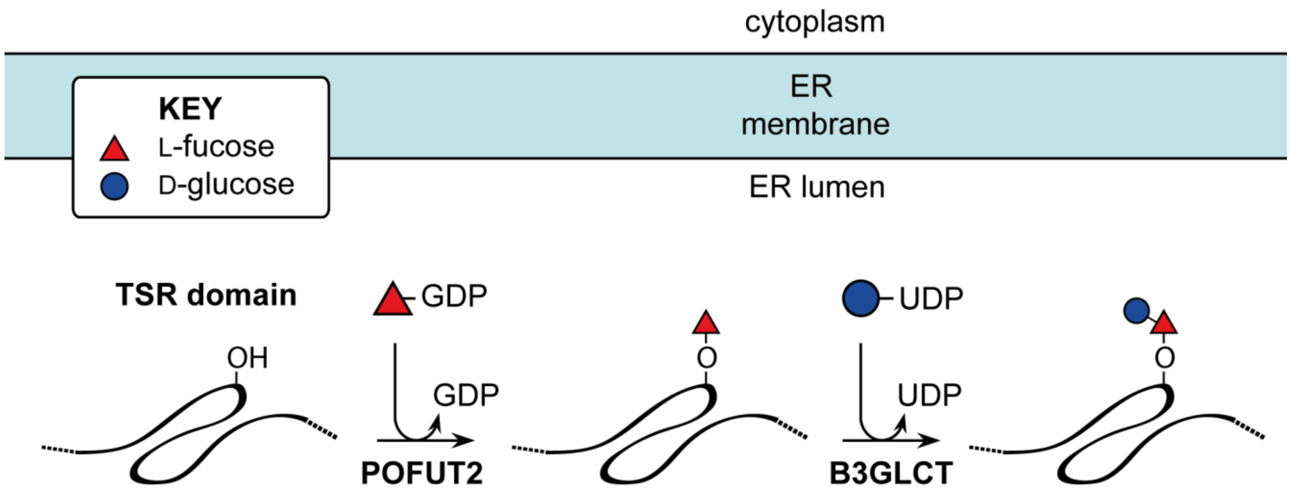

Supplementary Figure 1. O-glycosylation of TSR domains in metazoans.

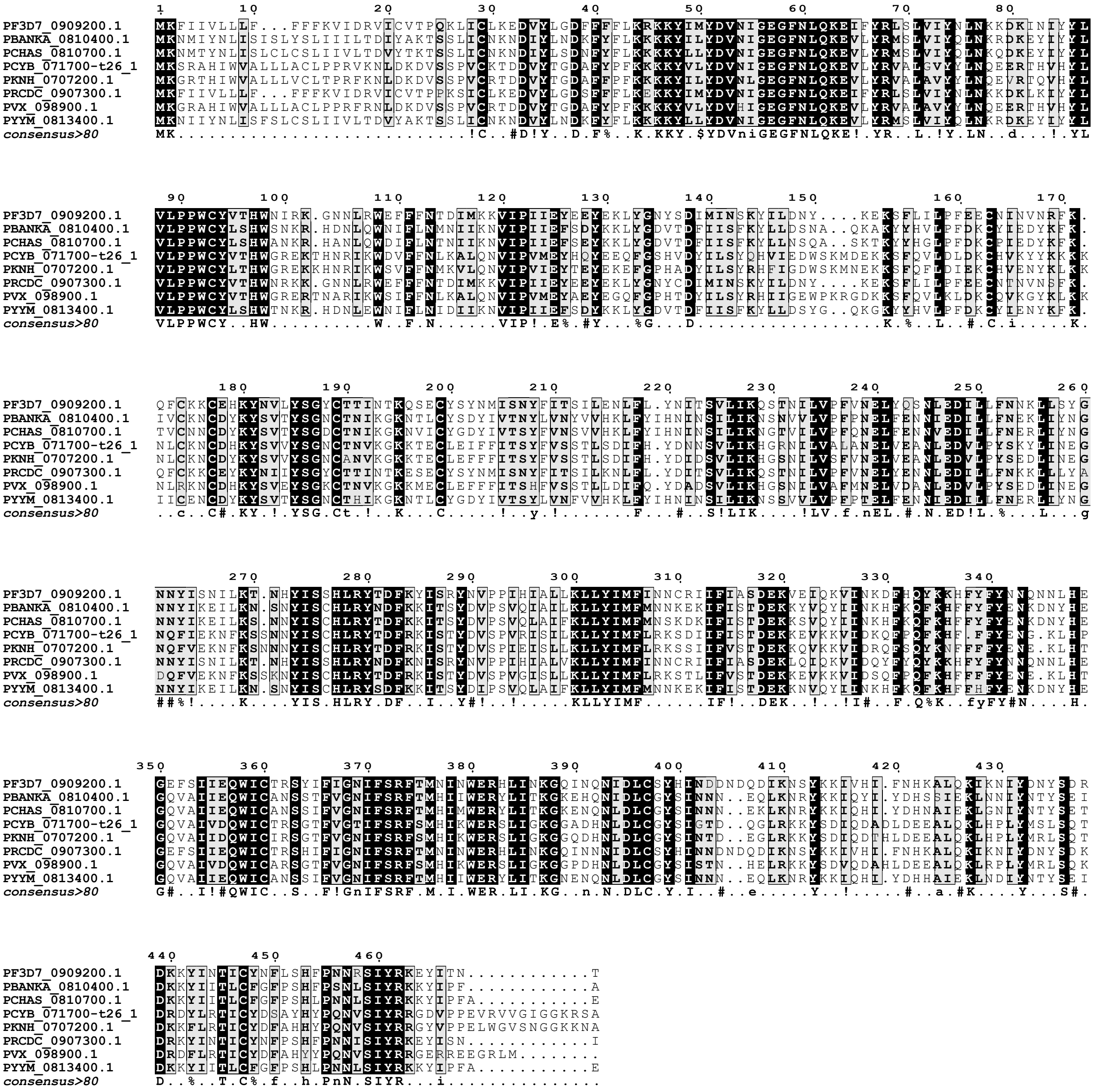

Supplementary Figure 2. Multiple sequence alignment of putative POFUT2s from *Plasmodium falciparum* (PF3D7_0909200.1), *P. berghei* (PBANKA_0810400.1), *P. chabaudi chabaudi* (PCHAS_0810700.1), *P. cynomolgi* (PCYB_071700-t26_1), *P. knowlesi* (PKNH_0707200.1), *P. reichenowi* (PRCDC_0907300.1), *P. vivax* (PVX_098900.1) and *P. yoelii yoelii* (PYYM_0813400.1). Black highlights indicate identity, gray highlights indicate similarity. For consensus sequence: uppercase is identity, lowercase is consensus level > 0.5, ! is anyone of IV, $ is anyone of LM, % is anyone of FY, # is anyone of NDQEBZ.

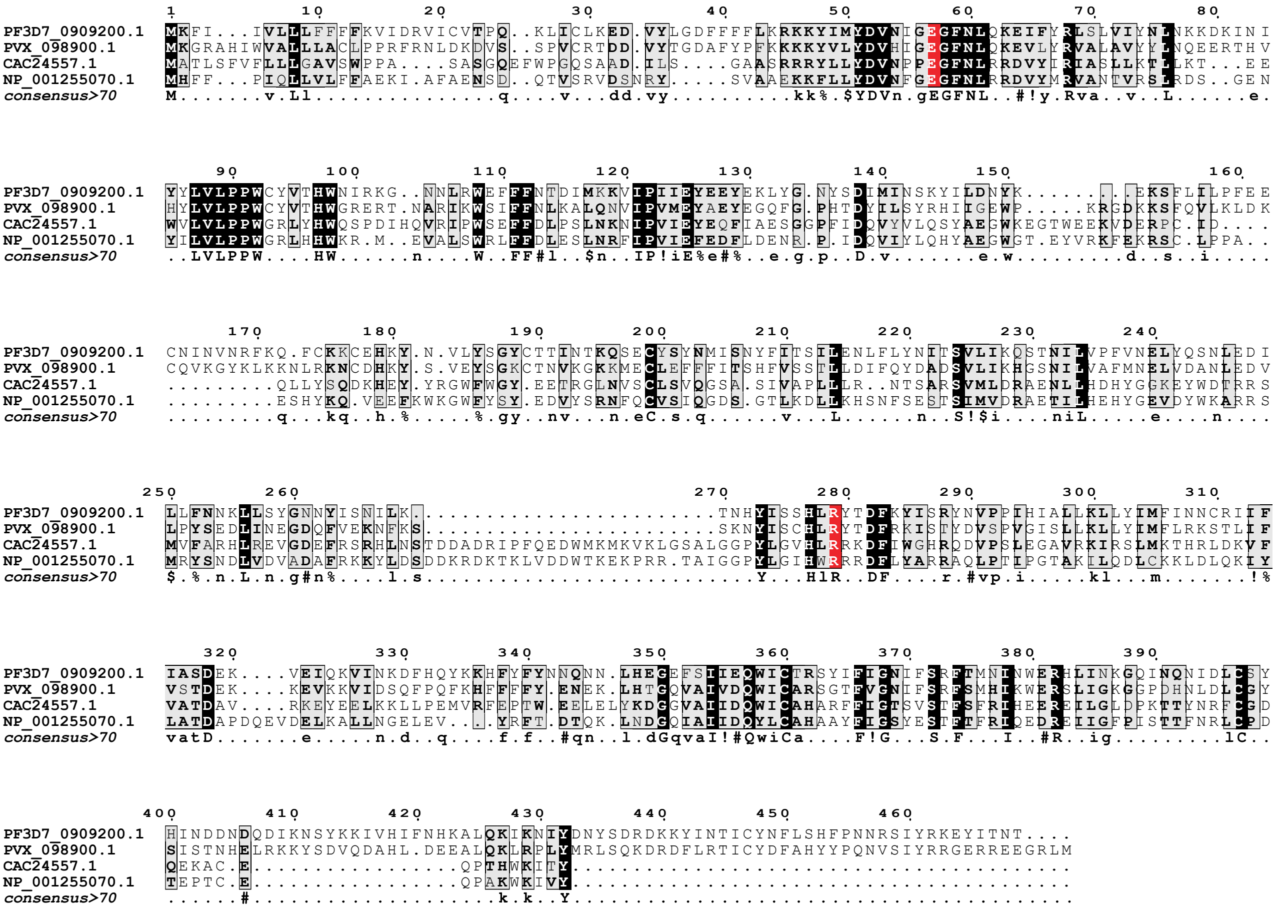

Supplementary Figure 3. Multiple sequence alignment of putative POFUT2s from *P. falciparum* (PF3D7_0909200.1) and *P. vivax* (PVX_098900.1) with POFUT2 from *Homo sapiens* (CAC24557.1) [1] and *Caenorhabditis elegans* (NP_001255070.1) [2]. The glutamate residue that serves as the catalytic base and the arginine residue that binds the pyrophosphate moiety of GDP-fucose, both highlighted in red, are conserved. Black highlights indicate identity, gray highlights indicate similarity. For consensus sequence: uppercase is identity, lowercase is consensus level > 0.5, ! is anyone of IV, $ is anyone of LM, % is anyone of FY, # is anyone of NDQEBZ.

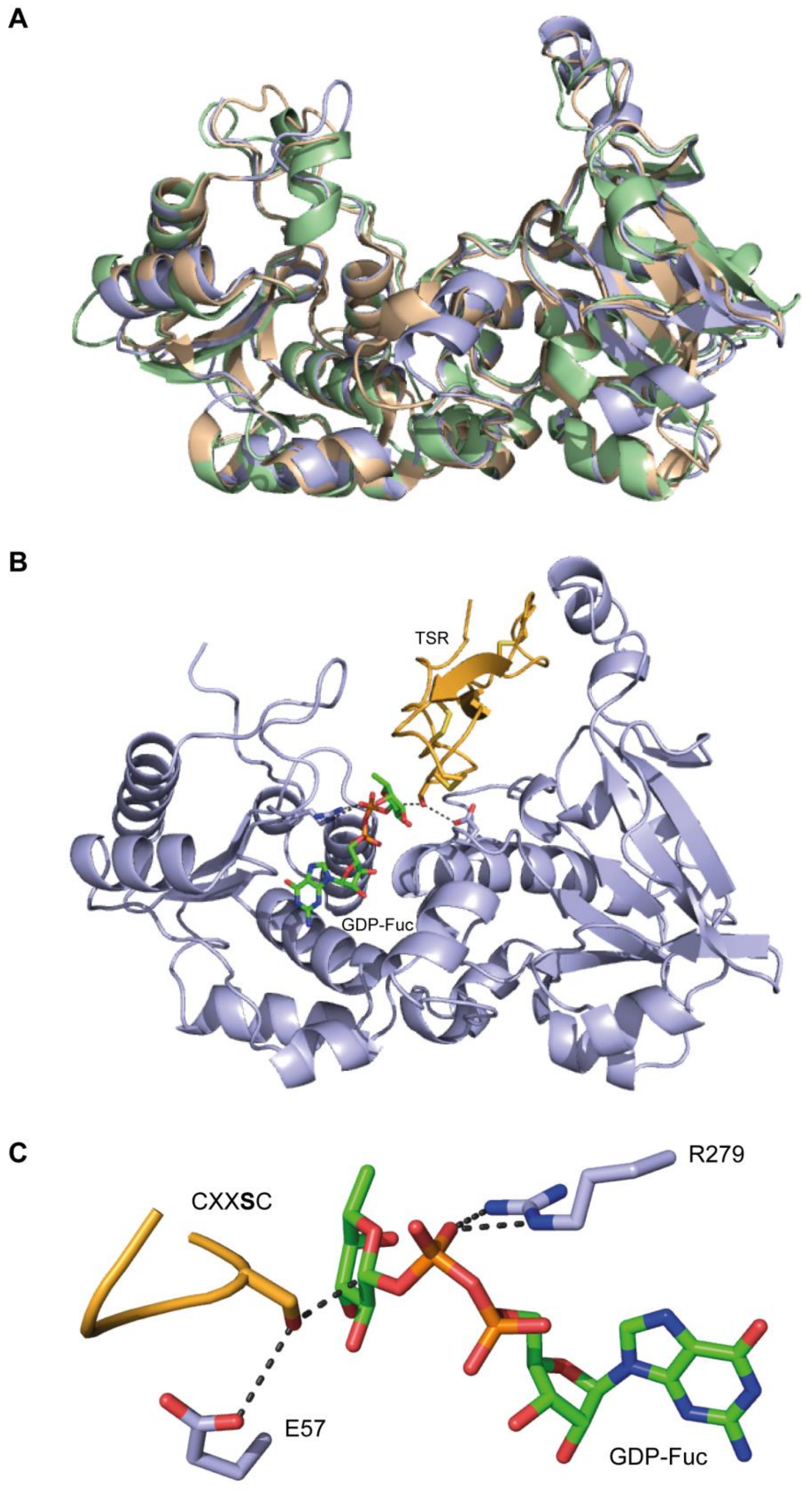

Supplementary Figure 4. (A) Homology model of *P. falciparum* POFUT2 (light blue) overlaid on the structures of *H. sapiens* POFUT2 (brown, 4AP5, RMS = 0.39 Å) [1] and *C. elegans* POFUT2 (pale green, 5FOE, RMS = 1.4 Å) [2], and (B) with GDP-fucose (green) and a TSR domain (gold) docked to give a plausible representation of the Michaelis complex. (C) The relative positions of substrates and catalytic residues within the active site of the *P. falciparum* POFUT2 homology model.

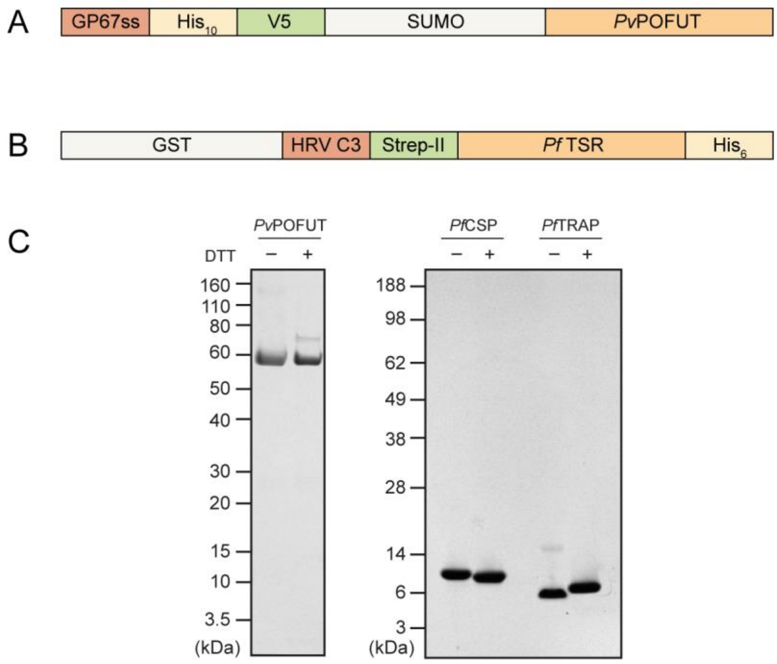

Supplementary Figure 5. (A) Construct design for expression of *P. vivax* POFUT2 using baculovirus in Sf21 insect cells. (B) Construct design for expression of *P. falciparum* CSP and TRAP TSR domains in *E. coli*. (C) Coomassie-stained SDS-PAGE gels of reduced and non-reduced samples of purified recombinant proteins.

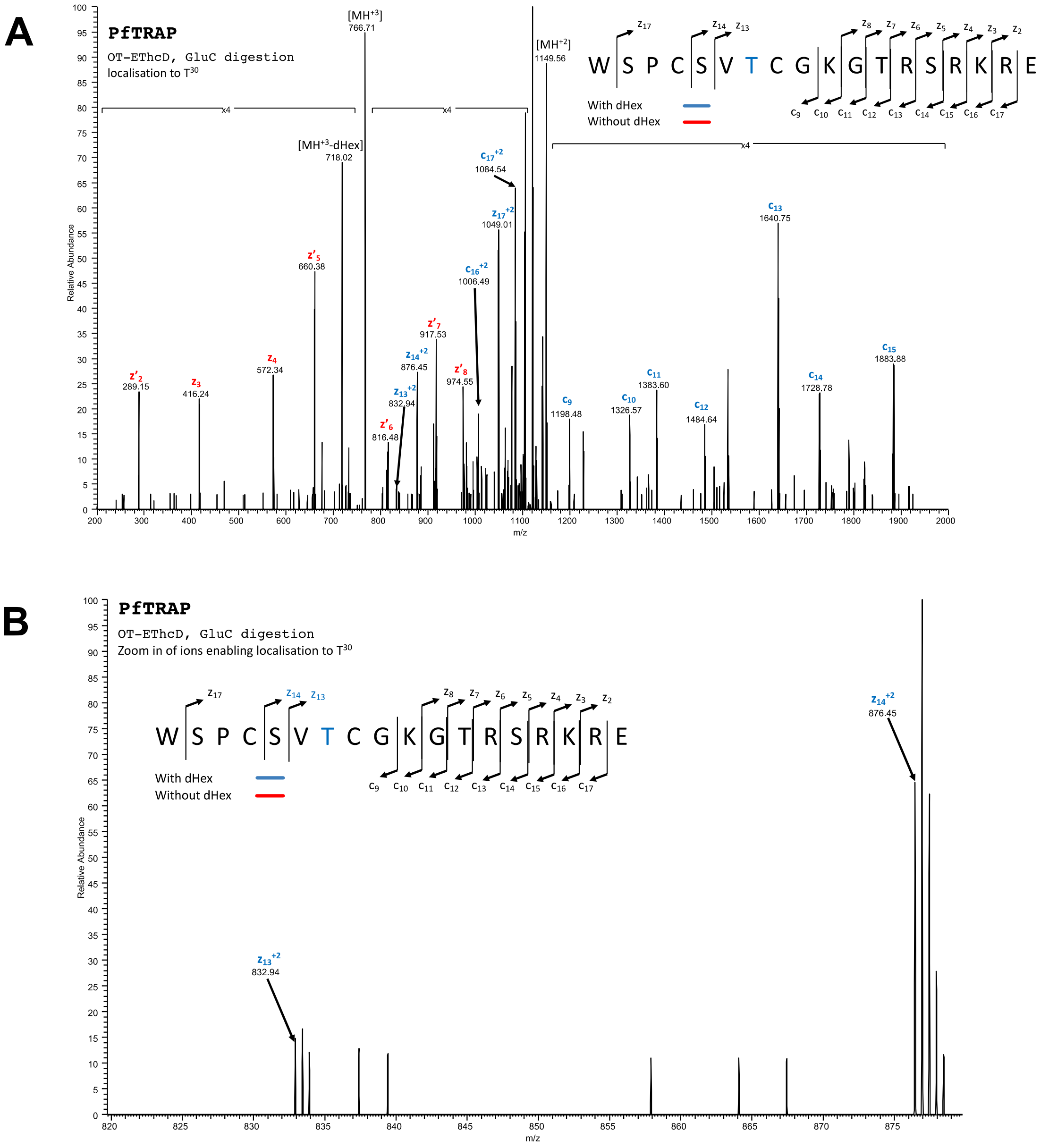

Supplementary Figure 6. LC-MS/MS analysis of GluC-digested PfTRAP that had been fucosylated *in vitro* by PvPOFUT2 enabled identification of the glycopeptide ^{24}WSPCSVTCGKGTRSRKRE^{41} (m/z= 766.70, z=+3). (A) EThcD fragmentation enabled localization of the fucosylation event to T^{30}. (B) Zoomed in region showing the z^{13} and z^{14} ion that definitively localize the glycosylation site to T^{30}.

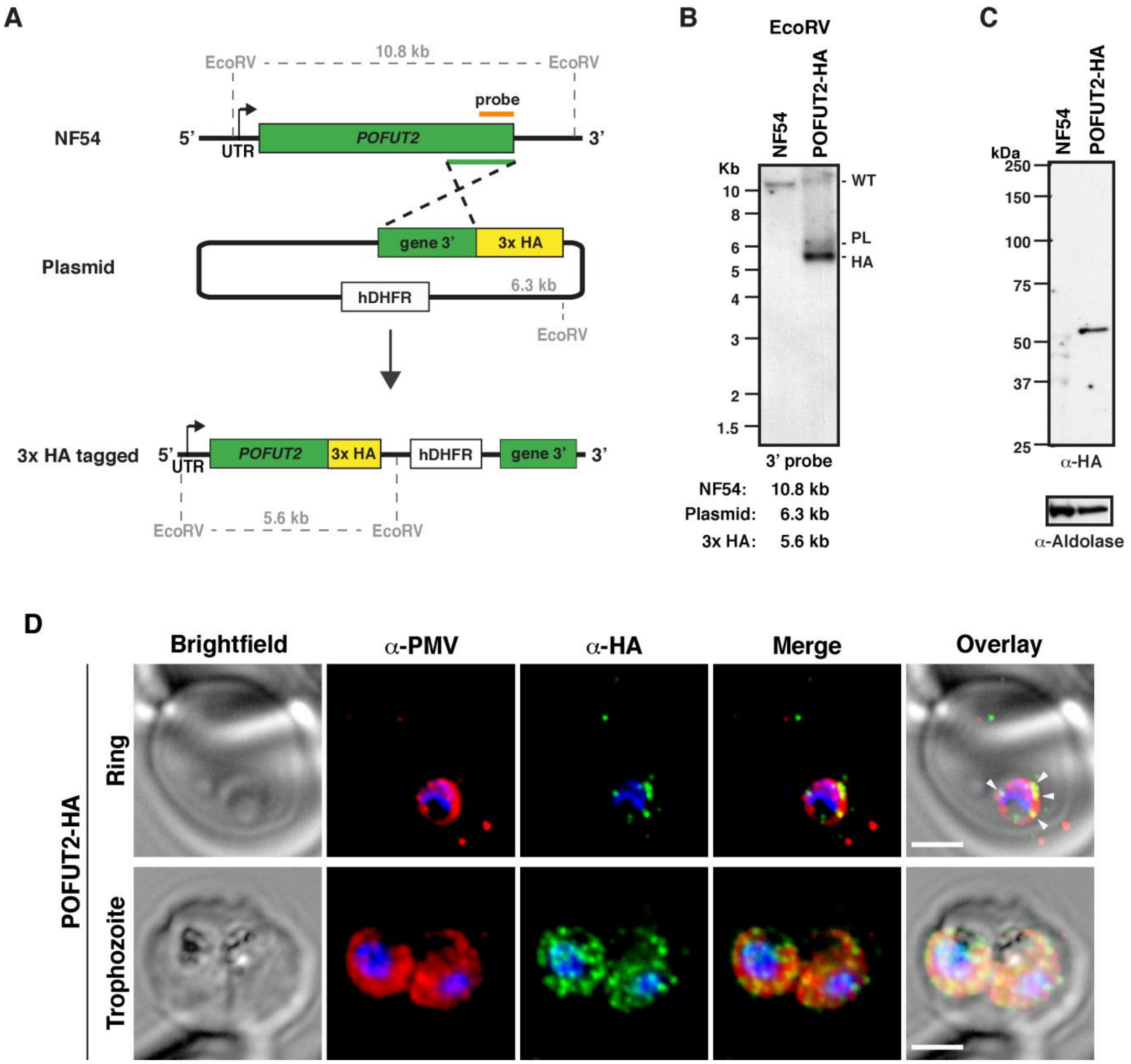

Supplementary Figure 7. (A) Strategy for inserting a C-terminal triple hemagglutinin (HA) epitope tag into the *P. falciparum* POFUT2 locus. (B) Southern blot analysis of parental NF54 and POFUT2-HA$_3$ parasite cultures. (C) Western blot analysis of NF54 and POFUT2-HA$_3$ trophozoites and schizonts. Anti-PfAldolase was used as a loading control. (D) Immunofluorescence microscopy of ring (top) and trophozoite (bottom) stage POFUT2-HA$_3$ parasites stained for the ER-resident protein plasmepsin V (PMV, red) and POFUT2-HA$_3$ (green) with the nucleus visualized using DAPI (blue). Punctae of co-localization (yellow) are evident in both micrographs and indicated with arrowheads in the ring parasite for reference. Scale, 2 μm.

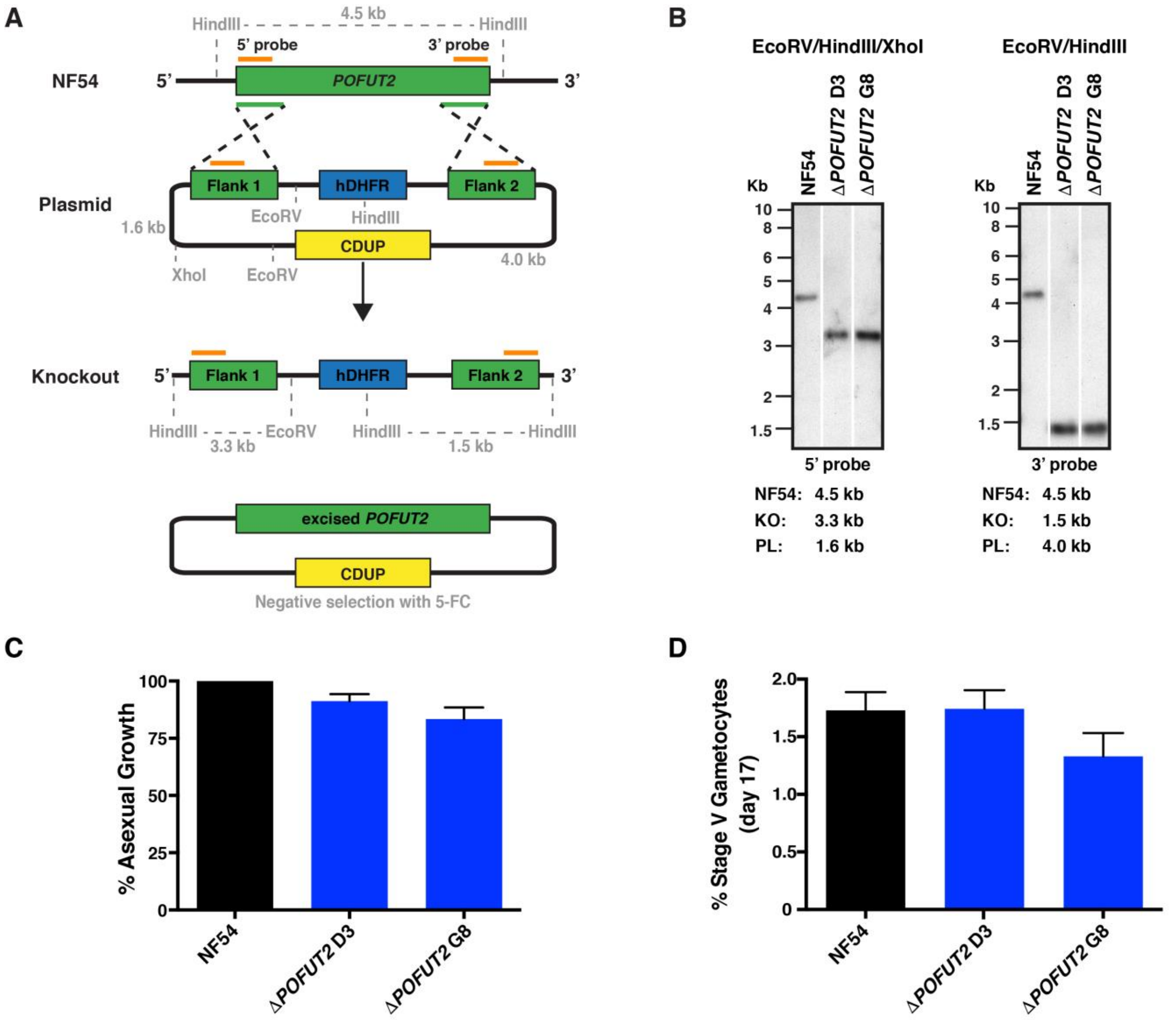

Supplementary Figure 8. (A) Strategy for disrupting the *POFUT2* locus in *P. falciparum* NF54. (B) Southern blot analysis of parental NF54 and two clonal Δ*POFUT2* parasite cultures (D3 and G8). (C) Quantitation of the blood stage growth rate of Δ*POFUT2* (D3 and G8) parasite cultures relative to NF54. (D) Quantitation of gametocytogenesis for NF54 and Δ*POFUT2* (D3 and G8) parasite cultures. Data in (C) and (D) are mean ± S.E.M from three independent experiments. No statistical differences were found relative to NF54 in panel (C) (p=0.2367) or panel (D) (p=0.1762) using the Kruskal-Wallis one-way ANOVA.

Supplementary Table 1. Oligonucleotides used in this study.

**Oligonucleotide**	**Sequence (5' to 3')***
PFOUTHA_F	ATCGGATCCCTTATTTTACCTTTCGAAGA
POFUTHA_R	ATCCTGCAGGTGTGTTGGTAATATATTCCT
POFUT2KO_FL1_F	ATCCCGCGGCTCTCAATTTGCGAAGCACA
POFUT2KO_FL1_R	GATACTAGTAACACCAAGGTGGAAGTACAAGA
POFUT2KO_FL2_F	ATCGAATTCTGTTCCTCCTATTCATATCGCTTT
POFUT2KO_FL2_R	GATCCTAGGTGTGTTGGTAATATATTCCTTTCG
POFUT2hm_F	TTCTTATTTTACCTTTCGAAGAATG
POFUT2hm_R	TGAATAACATTCACTCTGTTTTGTG
hDHFRhm_F	ACCTAATAGAAATATATCAGGATCC
hDHFRhm_R	GGTTGTGGTCATTCTCTGGA
Pf18S_F	GTAATTGGAATGATAGGAATTTACAGGT
Pf18S_R	TCAACTACGAACGTTTTAACTGCAAC
Pfs25_F	CAGATGAGTGGTCATTTGGAA
Pfs25_R	CAAGCGTATGAAACGGGATT
PfCTRP_F	GAATGGAGTCCCTGTCCTGA
PfCTRP_R	TGGTCCTTTCCTTTCCCTTT
Asrps7_F	TGCGGCTTCAGATCCGAGTTC
Asrps7_R	TTCGTTGTGAACCCAAATAAAAATC
PfTRAP_F	GTGTTTGGGACGAATGGTCT
PfTRAP_R	CGGGTTCATCTGGAACATCT
PfCSP_F	TGGGAAACAGGAAAATTGGT
PfCSP_R	CATCCGCTGGTTGCTTTAAT

*Restriction sites used for cloning are underlined.

Supplementary Methods

Generating PfPOFUT2 homology model

A homology model for *P. falciparum* POFUT2 was built using the Phyre2 server [3]. This model was overlaid on the structures of *H. sapiens* POFUT2 (4AP5) [1] and *C. elegans* POFUT2 (5FOE) [2] using PyMOL. The *P. falciparum* POFUT2 model, GDP-fucose and the structure of the TSR domain from 5FOE were docked together using the ROSIE server [4-6]. All images for Supplementary Figure 4 were generated using PyMOL.

P. falciparum TSR domain expression and purification

Codon-harmonised dsDNA polynucleotides encoding residues 242–288 of *P. falciparum* TRAP (PF3D7_1335900) and residues 307–376 of *P. falciparum* CSP (PF3D7_0304600) with an N-terminal Strep-II tag and C-terminal hexahistidine tag were synthesized (Integrated DNA Technologies) and cloned into pGEX6P3 (Invitrogen) at the BamHI and NotI restriction sites to give pGEX-PfTRAP and pGEX-PfCSP.

```
>tags-PfTSR
GGATCCTGGTCACATCCGCAGTTTGAAAAAGGTAGCGCAAGCTGTGGTGTTTGGGATGAATGGTCACCGTGTAG
CGTTACCTGTGGTAAAGGCACCCGTAGCCGTAAACGTGAAATTCTGCATGAAGGTTGTACCAGCGAACTGCAAG
AACAGTGTGAAGAAGAACGTTGTCTGCCTGGTAGCCATCATCATCACCATCATTAAGCGGCCGC

>tags-PfCSPTSR
GGATCCTGGTCACATCCGCAGTTTGAAAAAGGTAGCAATAATGAAGAACCGAGCGATAAACACATCAAAGAATA
TCTGAACAAAATCCAGAATAGCCTGAGCACCGAATGGTCACCGTGTAGCGTTACCTGTGGTAATGGTATTCAGG
TTCGTATTAAACCGGGTAGCGCAAATAAACCGAAAGATGAACTGGATTATGCCAACGATATCGAGAAAAAAATC
TGCAAAATGGAAAAATGCAGCAGCGTTGGTAGCCATCATCATCACCATCATTAAGCGGCCGC
```

Sequence-verified plasmids were transformed into *E. coli* BL21(DE3) and grown in 1.0 L LB media (100 µg ml^{-1} Amp) with shaking (250 rpm) at 37 °C until the culture reached an OD_{600} of 0.8. The culture was cooled to room temperature, IPTG added to a final concentration of 200 µM, and incubated with shaking (250 rpm) at 18 °C for 16 h. Cells were harvested by centrifugation (17,000 × g, 20 min, 4 °C) and the pellet was resuspended in 40 ml of binding buffer (50 mM Tris, 500 mM NaCl, 5 mM imidazole, pH 8) containing protease inhibitor (Roche complete EDTA-free protease inhibitor cocktail) and lysozyme (0.1 mg/ml) by nutating at 4°C for 30 min. Benzonase (1 µl, 25 U) was added to the mixture and lysis was effected by sonication. The lysate was centrifuged (17000 × g, 20 min, 4 °C) and the supernatant collected, filtered (0.45 µm) and loaded onto a 1 ml gravity IMAC column (GE HiTrap TALON). The column was washed with 15 ml binding buffer, then the protein was eluted using elution buffer (50 mM Tris, 500 mM NaCl, 500 mM imidazole, pH 8). Fractions containing product (as determined by SDS-PAGE) were combined and further purified by size exclusion chromatography (GE HiLoad *16/600 Superdex 200*) using 50 mM Tris,

150 mM NaCl, pH 7.5 buffer. Fractions containing monomeric product (as determined by SDS-PAGE on non-reduced samples) were concentrated (5 mg ml^{-1}) and incubated with HRV 3C protease (50 µg ml^{-1}), 1 mM EDTA, 2 mM reduced glutathione and 1 mM oxidized glutathione at 4 °C for 16 h. The liberated TSR domains were purified by size exclusion chromatography (GE HiLoad *16/600 Superdex 75* using 50 mM Tris, 150 mM NaCl, pH 7.5 buffer) and ion exchange chromatography (GE MonoQ 5/50 GL using 20 mM Tris, pH 7.5 with a NaCl gradient).

P. vivax POFUT2 expression and purification

A codon-harmonised dsDNA polynucleotide encoding residues 22–481 of *P. vivax* POFUT2 (PVX_098900) with an N-terminal gp67 signal peptide, His_{10} purification / V5 epitope tag, and SUMO fusion partner was synthesized (Integrated DNA Technologies) and cloned into pFastBac (Invitrogen) at the RsrII and XhoI restriction sites to give pFastBac-SUMOPvPOFUT2.

```
>SS-tags-SUMO-PvPOFUT
CGGTCCGAAACCATGCTACTAGTAAATCAGTCACACCAAGGCTTCAATAAGGAACACACAAGCAAGATGGTAAG
CGCTATTGTTTTATATGTGCTTTTGGCGGCGGCGGCGCATTCTGCCTTTGCGGCGGATCTTGGATCCCATCACC
ACCACCACCATCACCATCATCACGGTAAACCGATACCAAATCCCCTGCTCGGCCTCGACTCGACTATCGACGGT
CGCTCCGATTCCGAAGTAAACCAGGAGGCCAAACCTGAGGTCAAACCGGAAGTGAAACCCGAAACTCACATCAA
CTTGAAGGTCAGCGACGGCAGTTCGGAAATCTTCTTCAAAATCAAAAAAACTACCCCTTTGCGTAGACTTATGG
AAGCTTTCGCTAAGCGTCAGGGTAAAGAGATGGACTCCCTGACCTTTCTGTACGATGGAATTGAAATCCAGGCC
GACCAAACCCCTGAGGACCTCGACATGGAGGACAACGATATAATTGAGGCTCACCGCGAACAGATCGGTGGAGA
ATTCAATCTTGATAAGGATGTAAGTTCCCCCGTTTGTAGAACTGACGACGTATACACTGGAGACGCCTTTTACC
CTTTCAAGAAAAAGAAATACGTACTGTACGATGTGCATATCGGAGAGGGATTCAATCTTCAAAAGGAAGTTCTC
TACAGGGTCGCCCTTGCCGTGTACTATCTGAACCAGGAGGAACGCACACATGTGCACTACCTGGTACTCCCTCC
TTGGTGCTACGTTACGCACTGGGGACGTGAGAGGACTAACGCCAGGATCAAGTGGTCTATCTTTTTCAATCTGA
AAGCCCTGCAGAACGTAATCCCAGTTATGGAGTACGCGGAATACGAAGGACAGTTCGGCCCACATACAGATTAC
ATACTCTCGTACCGTCACATTATCGGCGAATGGCCCAAGCGTGGAGACAAGAAGAGCTTCCAGGTTCTCAAGTT
GGATAAGTGTCAAGTAAAGGGCTACAAGTTGAAGAAGAATCTGAGGAAGAACTGTGACCACAAGTACTCTGTTG
AGTATAGTGGAAAATGTACCAATGTCAAGGGCAAGAAAATGGAGTGCCTCGAATTTTTCTTTATTACATCACAT
TTCGTGTCCTCAACCCTTCTCGATATCTTCCAGTACGACGCTGATTCGGTGCTTATCAAGCACGGTAGTAACAT
CTTGGTGGCCTTCATGAACGAGCTTGTCGATGCTAATCTCGAAGACGTCCTGCCTTACTCCGAAGATCTGATCA
ACGAGGGCGACCAATTCGTCGAGAAAAATTTCAAGAGTTCCAAGAATTATATCAGCTGTCACCTTAGGTACACT
GATTTCCGCAAAATCTCCACTTACGACGTCAGCCCAGTGGGTATTAGCCTTCTGAAGCTGTTGTATATTATGTT
TCTGCGTAAGTCAACGTTGATCTTCGTCTCTACTGACGAAAAGAAGGAAGTGAAAAAAGTGATCGACAGCCAGT
TCCCTCAATTCAAGCATTTCTTCTTCTTCTACGAGAACGAAAAACTGCACACAGGACAAGTCGCAATAGTCGAC
CAGTGGATATGTGCACGCTCCGGAACATTCGTCGGCAACATTTTCAGTCGCTTCTCAATGCACATTAAGTGGGA
AAGATCCTTGATTGGAAAGGGCGGTCCAGATCACAACCTCGACTTGTGCGGCTACTCAATCTCGACTAATCACG
AACTGCGTAAAAAGTACAGTGATGTCCAAGACGCACATTTGGACGAGGAGGCTCTTCAGAAGCTGCGTCCACTC
TACATGCGCCTGTCTCAAAAAGACCGCGACTTCCTTAGGACCATCTGTTACGATTTCGCTCACTACTACCCACA
AAACGTCTCTATATACAGACGTGGAGAGAGGAGGGAGGAAGGCAGGTTGATGTGACTCGAG
```

Bacmid and baculovirus was prepared in accordance with the manufacturer’s instructions for the ‘Bac-To-Bac Baculovirus Expression System’. Sf21 cells (ThermoFisher Scientific #11497013, 1.8 L, 1.8×10^{6} cells/ml) were inoculated with P3 virus stock (200 ml) and incubated (27 °C, 110 rpm) for 72 h. The culture was centrifuged (300 × g, 5 min, 4 °C) and the supernatant filtered (0.22 µm), concentrated ten-fold (Amicon stirred cells, 10 kDa NMWL), dialysed (2×2.0 L of 50 mM Tris, 300 mM NaCl, pH 8.2) and loaded onto a 1 ml gravity IMAC column (GE HiTrap TALON). The

column was washed with 15 ml buffer (50 mM Tris, 300 mM NaCl, pH 8.2), then the protein was eluted using elution buffer (50 mM Tris, 300 mM NaCl, 500 mM imidazole, pH 8.2). Fractions containing product (as determined by SDS-PAGE) were combined and further purified by immunoaffinity chromatography using anti-V5 tag antibody agarose (Abcam, ab1229).

Intact protein mass spectrometry

Protein samples were prepared for intact protein analysis using a modified SP3 clean-up procedure[7]. Briefly, samples were acidified with formic acid (final concentration 2%, 5 mM DTT, final volume 30 µl) and incubated for 1 h at RT with 3 µl of SP3 beads and an equal volume of acetonitrile. Proteins were precipitated on to beads overnight with 240 µl ice-cold acetone at –20 °C. Beads were pelleted via magnetic capture and washed with 200 µl of ice-cold 80% acetone three times. Proteins were eluted with ice-cold 80% formic acid and rapidly diluted with 270 µl H_2O to prevent acid induced modifications. Eluted samples were snap-frozen, dried by lyophilisation and stored at –20 °C. Intact analysis was performed using a Maxis II ETD UHR-QqTOF mass spectrometer (Bruker Daltonics, Bremen, Germany) equipped with a Captivespray source and NanoBooster infusing acetonitrile at 0.2 $ml.min^{-1}$ to enhance MS signal intensity. Protein samples were resuspended in 2% formic acid and immediately loaded onto a ProSwift RP-4H 200 mm × 0.1 mm column (Thermo Scientific) and washed with buffer A (3% acetonitrile, 0.1% formic acid) for 10 min and then eluted with a linear gradient from 3–85% buffer B (99.9% acetonitrile, 0.1% formic acid) over 35 min at a flow rate of 1 $\mu l.min^{-1}$. MS1 Mass spectra were acquired at 1 Hz between a mass range of 150–2200 *m/z*. Intact mass analysis and deconvolution was performed using DataAnalysis 4.3 (Bruker).

Glycosylation site localization by mass spectrometry

Digestion of O-fucosylated TRAP: In vitro O-fucosylation samples were resuspended in 6 M urea, 2 M thiourea, 40 mM NH_4HCO_3 and reduced then alkylated[8] before being diluted 10-fold into 100 mM ammonium bicarbonate prior to the addition of GluC (1/50 w/w, Promega) and incubation overnight at 25°C. Digested samples were acidified to a final concentration of 0.5% formic acid and desalted using C_{18} stage tips [9,10] before analysis by LC-MS.

MS-MS Identification of O-fucosylation using reversed phase LC-MS: Purified peptides were re-suspended in buffer (2% acetonitrile, 0.1% TFA) and separated using a two-column chromatography set up composed of a PepMap100 C_{18} 20 mm × 75 µm trap and a PepMap C_{18} 500 mm × 75 µm analytical column (ThermoScientific). Samples were concentrated onto the trap

column (5 μl.min^{-1}) with buffer A (2% acetonitrile, 0.1% formic acid) for 5 min and infused into an Orbitrap Fusion™ Lumos™ Tribrid™ mass spectrometer (ThermoScientific) at 300 nl.min^{-1} via the analytical column using an Dionex Ultimate 3000 UPLC (ThermoScientific). Sixty min gradients were run altering the buffer composition from 1% to 28% buffer B (80% acetonitrile, 0.1% formic acid) over 35 min, then from 28% to 40% buffer B over 10 min, then from 40% to 100% buffer B over 2 min, the composition was held at 100% buffer B for 3 min, and then dropped to 3% buffer B over 5 min and held at 3% buffer B for another 10 min. The Lumos™ mass spectrometer was operated in a data-dependent mode automatically switching between the acquisition of a single Orbitrap MS scan (120,000 resolution) every 3 seconds and four different MS-MS approaches of the same precursor: HCD (Normalized collision energy of 35 with a maximum fill time of 80 ms or AGC of 2×10^5 analyzed within the Orbitrap at a resolution of 30K, EThcD (Predictive ETD reaction times were enabled [11] and a normalized collision energy of 25 set, with a maximum fill time of 120 ms or AGC 2×10^5 analyzed within the Orbitrap at a resolution of 30K), CID (Normalized collision energy of 30 with a maximum fill time 80 ms or AGC 2×10^5 using Ion trap based detection) and ETD (Predictive ETD reaction times were enabled [11] with a maximum fill time of 120 ms or AGC 2×10^5 using Ion trap based detection).

Data Analysis: Identification of O-fucosylated peptides was accomplished using MaxQuant (v1.5.3.1) [12]. Searches were performed against the amino acid sequence of the PfTRAP protein with GluC cleavage specificity allowing 2 miss cleavage events and the variable modifications of oxidation of methionine, O-fucosylation addition to serine or threonine and acetylation of protein N-termini. The precursor mass tolerance was set to 20 parts-per-million (ppm) for the first search and 10 ppm for main search, with a maximum false discovery rate (FDR) of 1.0% set for protein and peptide identifications. The resulting outputs were processed within the Perseus (v1.4.0.6) [13] analysis environment to remove reverse matches and common proteins contaminates prior to further analysis.

Transgenic parasites

To HA-tag the C-terminus of *P. falciparum* POFUT2, the 3′ of the *POFUT2* locus was amplified from *P. falciparum* NF54 gDNA using POFUT2HA_F and POFUT2HA_R primers. The amplicon was cloned into p1.2-SHA [14] via BamHI/PstI. Purified plasmid DNA (80 μg, Life Technologies) was transfected into *P. falciparum* NF54 and stable transfectants selected as described previously [15]. Integration of the cassette was confirmed by Southern blot analysis using the Roche digoxigenin (DIG) system according to the manufacturer's instructions.

To disrupt the *P. falciparum POFUT2* locus, 5′ and 3′ flanks were amplified from *P. falciparum* NF54 gDNA using primers POFUT2KO_FL1_F and POFUT2KO_FL1_R for flank 1 and POFUT2KO_FL2_F and POFUT2KO_FL2_R for flank 2. Primer sequences are provided in Supplementary Table 1. Flanks were cloned into pCC1 via SacII/SpeI (5′ flank) and EcoRI/AvrII (3′ flank) to make the knockout construct. Transfected lines were cloned by limiting dilution and analyses were performed on these clonal parasite lines as described above [16].

Supplementary References

1 Chen, C. I. *et al.* Structure of human POFUT2: insights into thrombospondin type 1 repeat fold and O-fucosylation. *EMBO J* **31**, 3183-3197, doi:10.1038/emboj.2012.143 (2012).

2 Valero-Gonzalez, J. *et al.* A proactive role of water molecules in acceptor recognition by protein O-fucosyltransferase 2. *Nat Chem Biol* **12**, 240-246, doi:10.1038/nchembio.2019 (2016).

3 Kelley, L. A., Mezulis, S., Yates, C. M., Wass, M. N. & Sternberg, M. J. The Phyre2 web portal for protein modeling, prediction and analysis. *Nature protocols* **10**, 845-858, doi:10.1038/nprot.2015.053 (2015).

4 Lyskov, S. & Gray, J. J. The RosettaDock server for local protein-protein docking. *Nucleic Acids Res* **36**, W233-238, doi:10.1093/nar/gkn216 (2008).

5 Lyskov, S. *et al.* Serverification of molecular modeling applications: the Rosetta Online Server that Includes Everyone (ROSIE). *PLoS One* **8**, e63906, doi:10.1371/journal.pone.0063906 (2013).

6 Combs, S. A. *et al.* Small-molecule ligand docking into comparative models with Rosetta. *Nature protocols* **8**, 1277-1298, doi:10.1038/nprot.2013.074 (2013).

7 Hughes, C. S. *et al.* Ultrasensitive proteome analysis using paramagnetic bead technology. *Mol Syst Biol* **10**, 757, doi:10.15252/msb.20145625 (2014).

8 Scott, N. E. *et al.* Simultaneous glycan-peptide characterization using hydrophilic interaction chromatography and parallel fragmentation by CID, higher energy collisional dissociation, and electron transfer dissociation MS applied to the N-linked glycoproteome of Campylobacter jejuni. *Mol Cell Proteomics* **10**, M000031-MCP000201, doi:10.1074/mcp.M000031-MCP201 (2011).

9 Ishihama, Y., Rappsilber, J. & Mann, M. Modular stop and go extraction tips with stacked disks for parallel and multidimensional Peptide fractionation in proteomics. *J Proteome Res* **5**, 988-994, doi:10.1021/pr050385q (2006).

10 Rappsilber, J., Mann, M. & Ishihama, Y. Protocol for micro-purification, enrichment, pre-fractionation and storage of peptides for proteomics using StageTips. *Nature protocols* **2**, 1896-1906, doi:10.1038/nprot.2007.261 (2007).

11 Rose, C. M. *et al.* A calibration routine for efficient ETD in large-scale proteomics. *J Am Soc Mass Spectrom* **26**, 1848-1857, doi:10.1007/s13361-015-1183-1 (2015).

12 Cox, J. & Mann, M. MaxQuant enables high peptide identification rates, individualized p.p.b.-range mass accuracies and proteome-wide protein quantification. *Nature biotechnology* **26**, 1367-1372, doi:10.1038/nbt.1511 (2008).

13 Tyanova, S., Temu, T. & Cox, J. The MaxQuant computational platform for mass spectrometry-based shotgun proteomics. *Nature protocols* **11**, 2301-2319, doi:10.1038/nprot.2016.136 (2016).

14 Boddey, J. A. *et al.* An aspartyl protease directs malaria effector proteins to the host cell. *Nature* **463**, 627-631, doi:10.1038/nature08728 (2010).

15 Yang, A. S. P. *et al.* Cell Traversal Activity is Required for *Plasmodium falciparum* Liver Infection in Humanized Mice. *Cell reports* **18**, 3105-3116, doi:10.1016/j.celrep.2017.03.017 (2017).

16 Sleebs, B. E. *et al.* Inhibition of Plasmepsin V activity demonstrates its essential role in protein export, PfEMP1 display, and survival of malaria parasites. *PLoS Biol* **12**, e1001897, doi:10.1371/journal.pbio.1001897 (2014).
